# Evolution of Highly Pathogenic Avian Influenza A(H5N1) Virus in Poultry, Togo, 2018

**DOI:** 10.3201/eid2512.190054

**Published:** 2019-12

**Authors:** Maxime Fusade-Boyer, Pidemnéwé S. Pato, Mathias Komlan, Koffi Dogno, Trushar Jeevan, Adam Rubrum, Casimir K. Kouakou, Emmanuel Couacy-Hymann, Daniel Batawui, Emilie Go-Maro, Pamela McKenzie, Richard J. Webby, Mariette F. Ducatez

**Affiliations:** Université de Toulouse, Toulouse, France (M. Fusade-Boyer, M.F. Ducatez);; Laboratoire Central Vétérinaire de Lomé, Lomé, Togo (P.S. Pato, M. Komlan, K. Dogno, D. Batawui, E. Go-Maro);; St. Jude Children’s Research Hospital, Memphis, Tennessee, USA (T. Jeevan, A. Rubrum, P. McKenzie, R.J. Webby);; Central Laboratory for Animal Diseases, Bingerville, Côte d’Ivoire (C.K. Kouakou, E. Couacy-Hymann)

**Keywords:** Africa, highly pathogenic avian influenza, influenza virus, Togo, Côte d’Ivoire, reassortment, genetic evolution, poultry, phylogeny, molecular clock, viruses, respiratory infections, H5N1, zoonoses

## Abstract

In 2015, highly pathogenic avian influenza A(H5N1) viruses reemerged in poultry in West Africa. We describe the introduction of a reassortant clade 2.3.2.1c virus into Togo in April 2018. Our findings signal further local spread and evolution of these viruses, which could affect animal and human health.

Relatively little is known about the emergence, prevalence, and circulation of animal influenza viruses in Africa. Highly pathogenic avian influenza (HPAI) H5N1 clade 2.2 viruses emerged in Africa in 2006 (cases were reported from Egypt, Nigeria, Côte d’Ivoire, Benin, Togo, Ghana, Sudan, Djibouti, and Cameroon), although the virus was only maintained long term in Egypt (*1*,*2*). We conducted surveillance in domestic poultry in Côte d’Ivoire, Benin, and Togo during 2008–2010 but were unable to find virologic or serologic evidence for influenza A virus circulation. Several factors, such as type of hosts, climate, and animal density, might have provided unfavorable conditions for the virus’ circulation in the region (*3*).

In early 2015, HPAI H5N1 viruses of clade 2.3.2.1c were reported in Nigeria (*4*), followed closely by detections in Burkina Faso, Côte d’Ivoire, Ghana, Niger, Cameroon, and Togo (*2*). These viruses could be clustered into 2 genetic subgroups (*5*); cluster WA1 viruses were detected in Ghana, Burkina Faso, Côte d’Ivoire, Nigeria, and Niger, and cluster WA2 viruses were detected in Niger, Côte d’Ivoire, and Nigeria (*6*,*7*). In this study, we aimed to determine the origin and evolution of HPAI A(H5N1) viruses responsible for poultry outbreaks in Togo in April 2018 in the context of viruses from surrounding countries.

## The Study

In April 2018, high mortality rates were reported in chickens (84%) and quails (27%) on a farm with 4,371 domestic birds and 89 swine in Lacs Province in the south of Togo. Necropsies revealed petechiae and hemmorrhages in tracheas, bursa of Fabricius, lungs, and livers. We suspected influenza A virus, which we subsequently confirmed by using the Flu Detect rapid test (Synbiotics Corporation, http://www.synbiotics.com) according to the manufacturer’s instructions. We collected 15 samples, including cloacal and tracheal swab specimens and tissues (liver, trachea, lung, and spleen), from euthanized chickens with clinical signs and confirmed the presence of H5 by using reverse transcription PCR. As a precaution, all the animals on the farm (including swine) were slaughtered, even if clinical signs were observed only in birds. Humans in contact with animals on the farm were put under surveillance by public health services (no samples were collected but medical checkup was offered).

To determine the relationship of the viruses from Togo with viruses from neighboring countries, we also analyzed samples collected during April 2015–October 2016 during HPAI A(H5N1) outbreaks in Côte d’Ivoire. We successfully isolated 15 viruses from Togo and 32 viruses from Côte d’Ivoire. We performed hemagglutination inhibition assays, as previously described (*8*); these assays indicated a similar antigenic profile of viruses from the 2 countries. The noticeable exception was that most viruses from Côte d’Ivoire reacted more robustly to antiserum derived from the 2.3.2.1a virus A/duck/Bangladesh/19097/2013 (Appendix Table). We obtained full hemagglutinin (HA) and neuraminidase (NA) gene segment sequences by using Sanger sequencing (GenBank accession nos. MK071279 and MK084618). These sequences clustered with clade 2.3.2.1c HPAI A(H5N1) viruses from western Africa when analyzed using maximum-likelihood phylogenies (Appendix Figures 1, 2).

All the isolates from Togo were closely related to each other, which can be explained by the limited duration of the outbreak, and clustered with WA2 2.3.2.1c viruses on the basis of their HA sequence but with WA1 2.3.2.1c viruses on the basis of their NA sequence; the highest similarities in both cases were to viruses from Nigeria. Such reassortants have been previously observed in Nigeria and Cameroon (*7*,*9*), providing further support for Nigeria (and not Côte d’Ivoire) as a possible source of the Togo virus. Although most similar to sequences from viruses in Nigeria, the sequences from Togo displayed a degree of divergence (1.3% as calculated with the maximum composite distance) from their closest relatives as evidenced by tree topologies (Appendix Figures 1, 2). On the basis of HA sequences, the time to the most recent common ancestor (tMRCA) of the Togo 2018 viruses was estimated as November 2017 (95% highest posterior density [HPD] interval May 2017–April 2018), as determined in a relaxed molecular clock method under the Bayesian Markov chain Monte Carlo framework in BEAST 1.7.1 (*10*) and implemented on a Galaxy workbench (http://galaxy-workbench.toulouse.inra.fr) with parameters previously described (*11*). The tMRCA of the viruses from Togo, Nigeria, and Cameroon was estimated as September 2015 (95% HPD interval August–November 2015), suggesting a gap in surveillance and sequence data in the region during 2015–2018 (Appendix Figure 3). Whether outbreaks went unnoticed or unreported in the region or whether specific selection pressures might have existed in Togo still requires further investigation.

We observed higher genetic diversity among the sequences of the 2015–2016 isolates from Côte d’Ivoire than among the isolates from Togo. Most viruses from Côte d’Ivoire that we sequenced in this study belonged to the subcluster WA1 (on the basis of their HA and NA gene segments) and were closely related to viruses from Burkina Faso, Nigeria, and Ghana (closest strain was A/domestic bird/Burkina Faso/15VIR1774-22/2015) (Appendix Figures 1, 2). Two isolates from Côte d’Ivoire clustered with WA2 viruses from the same outbreak from Côte d’Ivoire and viruses from Nigeria in both HA and NA phylogenies (Appendix Figures 1, 2). We also identified WA1 HA and WA2 NA reassortants. Full genome sequencing of these isolates from Togo and Côte d’Ivoire is warranted to allow for a full assessment of recent genetic reassortments in the region. 

On the basis of HA sequences, we estimated the tMRCA of the WA2 strains from Côte d’Ivoire (as well as those from the closely related strains previously reported from Nigeria and Côte d’Ivoire) as January 2015 (95% HPD interval July 2014–February 2015), and we estimated the tMRCA of the WA1 strains from Côte d’Ivoire (and their related counterparts from Nigeria and Burkina Faso) as February 2015 (95% HPD interval December 2014–March 2015) (Appendix Figure 3). Both the phylogeny and the molecular clock analyses show multiple introductions of HPAI A(H5N1) clade 2.3.2.1c viruses into Côte d’Ivoire during the 18 months of the outbreaks (April 2015–October 2016). Although we performed limited (and not randomized) sampling, which probably yielded results that are not representative of the complete epidemiologic context, we nevertheless observed substantial virus diversity in the region.

## Conclusions

After a period of absence, HPAI clade 2.3.2.1c H5 viruses have spread in sub-Saharan Africa in the past 3 years. A single introduction of virus into the region might have occurred, followed by local spread, leading to genetic and antigenic diversification. The high prevalence of these viruses in countries with large commercial poultry industries, such as Nigeria and Côte d’Ivoire, has resulted in reassortment between local viral lineages. Adding to the complexity, HPAI A(H5N8) viruses from clade 2.3.4.4 have recently been reported in Cameroon and Nigeria (*2*,*12*), and low-pathogenicity avian influenza H9N2 viruses have also spread from northern to western Africa in 2017–2018 (with a report from Burkina Faso) (*13*–*15*). If these viruses also establish endemicity, the avian influenza situation in western Africa will be in stark contrast to the situation over the past decade or more, when limited virus circulation occurred. The threat to animal and public health should therefore be seriously reconsidered, especially because veterinary services in the region might not operate at the efficiency required to quickly identify and contain outbreaks.

Appendix Additional information regarding evolution of highly pathogenic avian influenza A(H5N1) virus in poultry, Togo, 2018.
