# Supplementary material for: Evolution of Highly Pathogenic Avian Influenza A(H5N1) Virus in Poultry, Togo, 2018
Source: Emerg Infect Dis. 2019 Dec;25(12):2287–9. doi: 10.3201/eid2512.190054 (PMC6874233; doi:10.3201/eid2512.190054)
Supplement: Appendix — Additional information regarding evolution of highly pathogenic avian influenza A(H5N1) virus in poultry, Togo, 2018. [file 19-0054-Techapp-s1.pdf]

Article DOI: https://doi.org/10.3201/eid2512.190054

Evolution of Highly Pathogenic Avian Influenza A(H5N1) Virus in Poultry, Togo, 2018

Appendix

Appendix Table. Antigenic properties of selected Togo and Côte d'Ivoire HPAI A(H5N1) 2.3.2.1c isolates as measured by Hemagglutination Inhibition assay (<: HI titer <10).

Antigen or virus	H5N1 clade	Hubei/1	dk/Bd/19097	BS/HK/1161	dk/VN/NCVD	HK/6841	ck/Ghana/20	cm/HK/5052
Reference antigens								
rg-A/Hubei/1/2010 (IDCDC-RG30)	2.3.2.1a	320	160	320	320	320	80	80
rg-A/duck/Bangladesh/19097/2013 (SJ007)	2.3.2.1a	320	320	320	320	320	160	80
rg-A/barn swallow/Hong Kong/1161/2010 (SJ003)	2.3.2.1b	40	80	320	160	160	80	80
rg-A/duck/Vietnam/NCVD-1584/2012 (NIBRG-301)	2.3.2.1c	320	320	320	320	320	160	80
A/Hong Kong/6841/2010	2.3.2.1c	160	80	160	160	160	80	40
A/chicken/Ghana/20/2015	2.3.2.1c	<	20	<	10	40	80	20
rg-A/common magpie/Hong Kong/5052/2007 (SJRG-166615)	2.3.2.1c	40	80	320	80	160	320	160
Viruses from Togo								
A/chicken/Togo/504/2018	2.3.2.1c	<	<	<	10	40	80	10
A/chicken/Togo/505/2018	2.3.2.1c	<	10	<	20	40	80	10
A/chicken/Togo/506/2018	2.3.2.1c	<	10	<	10	20	40	<
A/chicken/Togo/507/2018	2.3.2.1c	<	10	<	10	20	40	<
A/chicken/Togo/508/2018	2.3.2.1c	<	10	<	20	40	80	10
A/chicken/Togo/509/2018	2.3.2.1c	<	10	<	20	40	80	10
A/chicken/Togo/510/2018	2.3.2.1c	<	40	10	20	80	160	20
A/chicken/Togo/511/2018	2.3.2.1c	<	10	<	10	40	80	10
Viruses from Côte d'Ivoire								
A/chicken/Côte d'Ivoire/008–8/2016	2.3.2.1c	<	20	<	10	20	NA	10
A/guinea fowl/Côte d'Ivoire/13–17/2016	2.3.2.1c	<	160	10	40	40	NA	<
A/goose/Côte d'Ivoire/12–38/2016	2.3.2.1c	<	320	20	160	160	NA	10
A/chicken/Côte d'Ivoire/001–100/2016	2.3.2.1c	<	160	10	40	40	NA	<
A/guinea fowl/Côte d'Ivoire/003–103/2016	2.3.2.1c	<	160	10	40	40	NA	<
A/chicken/Côte d'Ivoire/207–2/2015	2.3.2.1c	<	160	<	40	20	NA	<
A/chicken/Côte d'Ivoire/232–5/2015	2.3.2.1c	<	160	10	40	40	NA	10
A/chicken/Côte d'Ivoire/275–11/2015	2.3.2.1c	10	320	320	160	160	NA	80
A/chicken/Côte d'Ivoire/301–20/2015	2.3.2.1c	<	160	<	40	40	NA	<
A/guinea fowl/Côte d'Ivoire/274–44/2015	2.3.2.1c	<	160	160	80	80	NA	40
A/chicken/Côte d'Ivoire/225–71/2015	2.3.2.1c	<	80	<	40	40	NA	<
A/duck/Côte d'Ivoire/152–83/2015	2.3.2.1c	<	80	<	20	10	NA	<
A/chicken/Côte d'Ivoire/262–86/2015	2.3.2.1c	<	160	160	80	80	NA	40

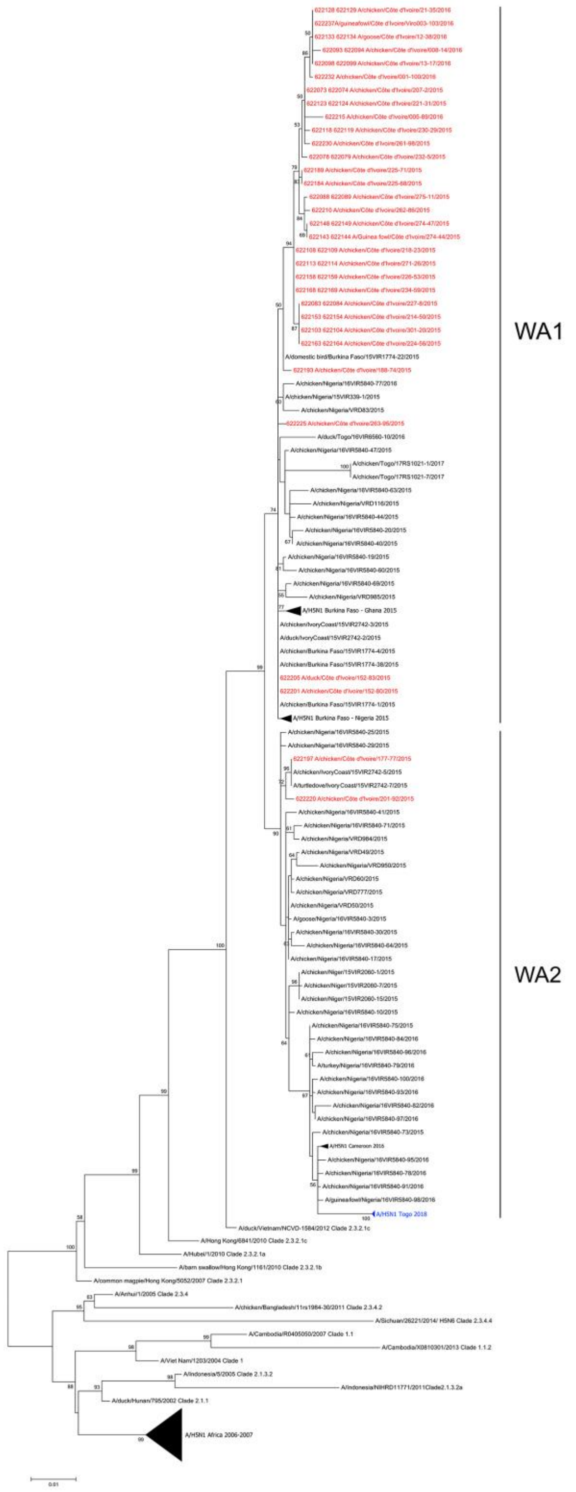

Appendix Figure 1. Hemagglutinin (HA) phylogenetic tree constructed by the maximum-likelihood method. A(H5N1) influenza viruses from Togo are labeled blue and those from Côte d'Ivoire, red. Bootstrap support higher than 50% is indicated above the nodes. Scale bar indicates the number of nucleotide substitutions per site.

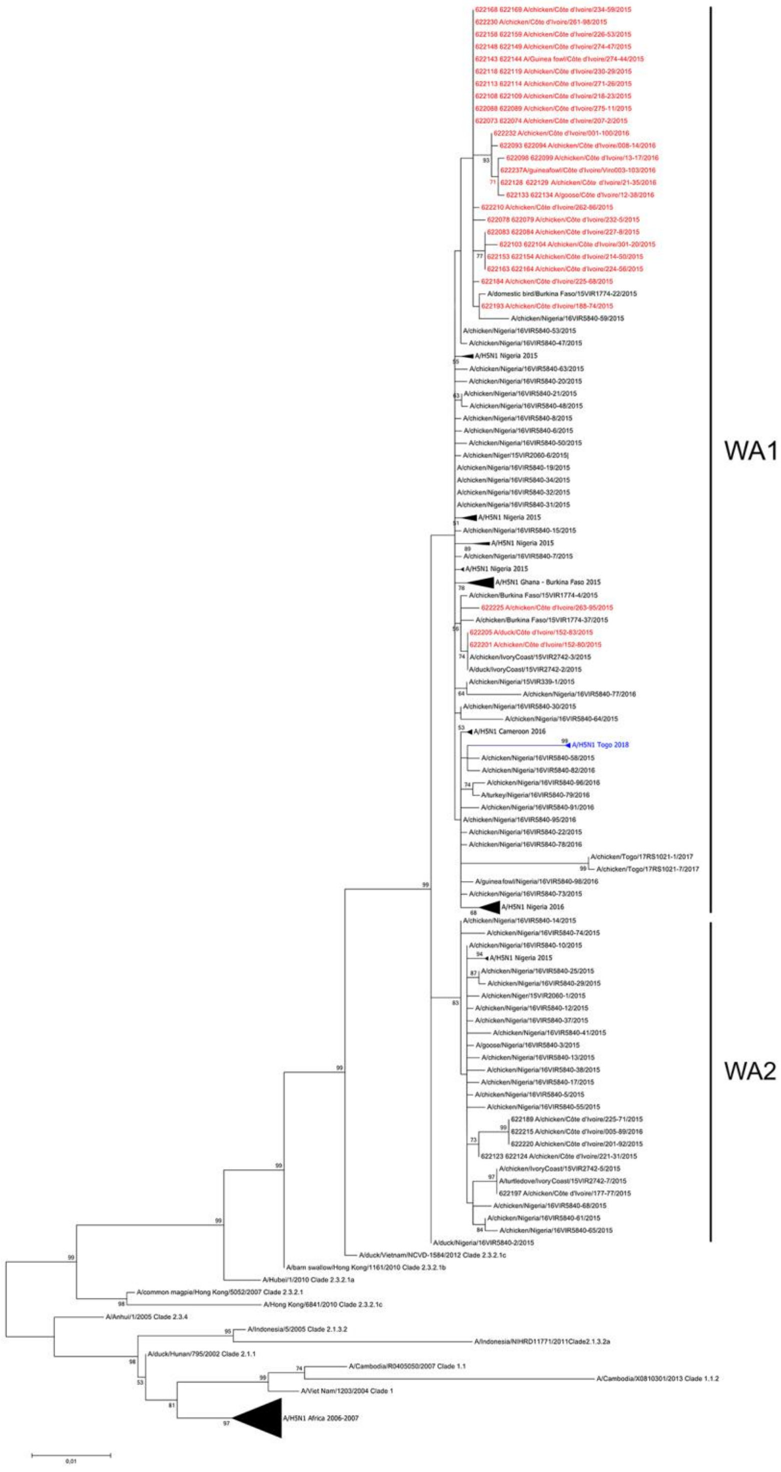

Appendix Figure 2. Neuraminidase (NA) phylogenetic tree constructed by the maximum-likelihood method. A(H5N1) influenza viruses from Togo are labeled blue and those from Côte d’Ivoire, red. Bootstrap support higher than 50% is indicated above the nodes. Scale bar indicates the number of nucleotide substitutions per site.

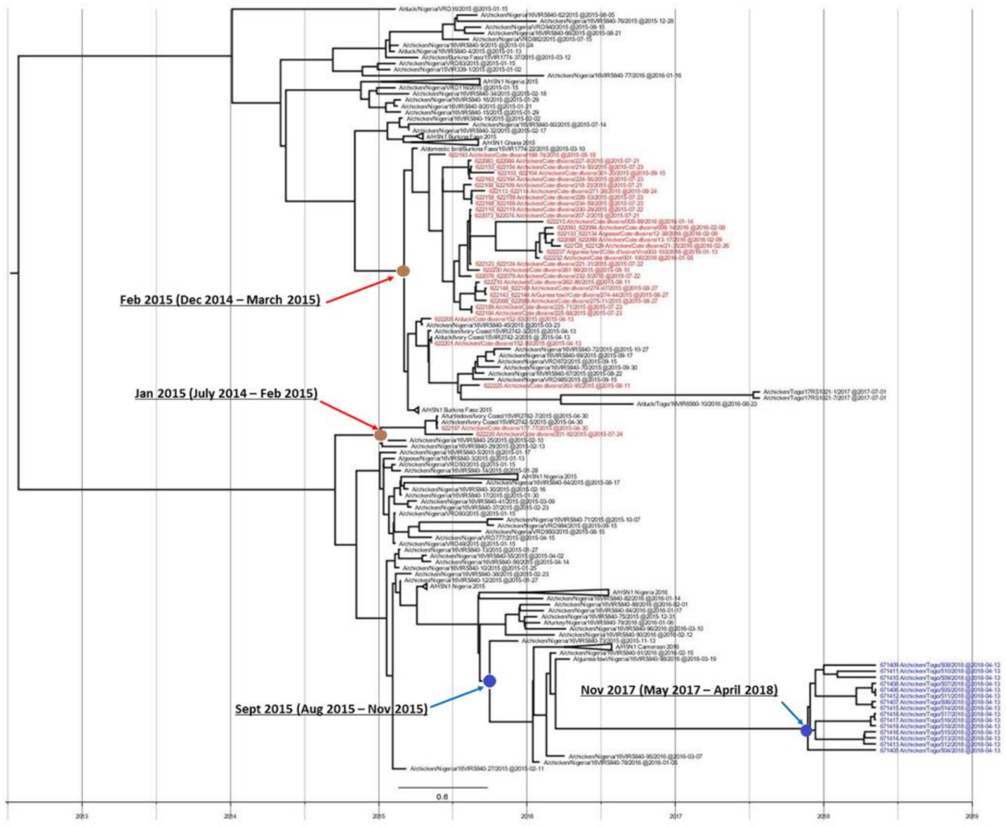

Appendix Figure 3. Maximum clade credibility phylogenetic tree of the hemagglutinin (HA) gene segment. The H5N1 viruses from Togo are represented in blue and those from Côte d'Ivoire are represented in red. The mean time to the most common ancestor (tMRCA) and the 95% highest posterior density intervals of the relevant nodes are indicated in parentheses.
